# Supplementary material for: Causal Relationship between Aging and Anorexia Nervosa: A White-Matter-Microstructure-Mediated Mendelian Randomization Analysis
Source: Biomedicines. 2024 Aug 16;12(8):1874. doi: 10.3390/biomedicines12081874 (PMC11351342; doi:10.3390/biomedicines12081874)
Supplement: Supplementary file 1 [file biomedicines-12-01874-s001.zip › Supplementary Figure/Figure S8/Leaveoneout plot of MR analysis for TL to ubm-b-1761.pdf]

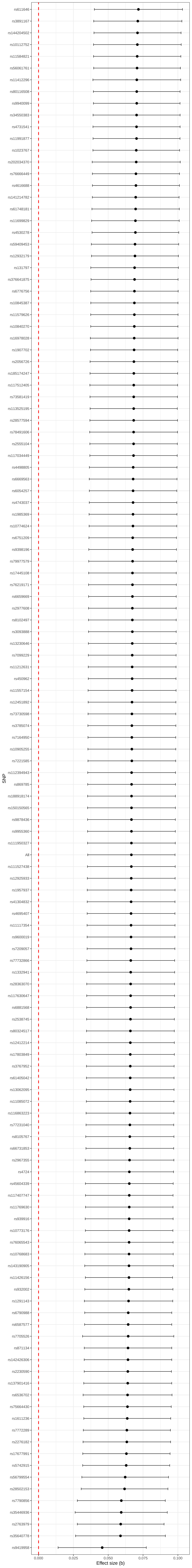

rs611646
rs3891167
rs144204502
rs10127752
rs11584821
rs56061761
rs11412296
rs80116508
rs9940099
rs34550383
rs4731541
rs11991877
rs1023767
rs202034370
rs76666449
rs4616688
rs141214782
rs61748181
rs11699829
rs4530278
rs59409453
rs12932179
rs131797
rs376641875
rs6776756
rs10845387
rs11579626
rs10840270
rs16978028
rs1907702
rs2056726
rs185174247
rs117512405
rs73581419
rs113525195
rs28577594
rs78491606
rs2555104
rs117034449
rs4498805
rs6669563
rs6054257
rs4743037
rs1985369
rs10774624
rs6751209
rs9398196
rs79977579
rs17445108
rs76219171
rs6659669
rs2977608
rs8102497
rs3093888
rs13230646
rs7099229
rs11212631
rs450962
rs11557154
rs12451892
rs73730598
rs3785074
rs7164950
rs10905255
rs7221585
rs112394943
rs869785
rs188918174
rs150150565
rs9878436
rs9955360
rs111950327
All
rs111527438
rs12925933
rs1957937
rs41304832
rs4695407
rs11117354
rs9600019
rs7209057
rs77732866
rs1332941
rs28363070
rs117630647
rs6881568
rs2538745
rs80324517
rs12412214
rs17803849
rs3767952
rs61405042
rs13062095
rs11085072
rs116863223
rs77231040
rs8105767
rs66731853
rs2967355
rs4724
rs45604339
rs117407747
rs11769630
rs939916
rs10773176
rs76065543
rs10768683
rs143190905
rs11426156
rs932002
rs1291143
rs6790988
rs6587577
rs7705526
rs871134
rs142426306
rs2230590
rs137901416
rs6536702
rs75664430
rs1611236
rs7772289
rs2276182
rs17677991
rs5742915
rs56799554
rs28502153
rs7790856
rs35446936
rs2763979
rs35640778
rs9419958
SNP
0.000
0.025
0.050
0.075
0.100
Effect size (b)
